# Larotrectinib in TRK fusion differentiated thyroid carcinoma: updated trial data

**DOI:** 10.1530/ERC-25-0531

**Published:** 2026-08-05

**Authors:** Steven G Waguespack, Marcia S Brose, Jessica J Lin, Ray McDermott, Mohammed Almubarak, Jessica Bauman, Michela Casanova, Shivaani Kummar, Se-Hoon Lee, Damian T Rieke, Do-Youn Oh, Changsong Qi, Natascha Neu, Domnita-Ileana Burcoveanu, Chiara E Mussi, Alexander Drilon, David S Hong, Maria E Cabanillas

**Affiliations:** ^1^ Department of Endocrine Neoplasia and Hormonal Disorders, The University of Texas MD Anderson Cancer Center, Houston, Texas, USA; ^2^ Department of Medical Oncology, Sidney Kimmel Comprehensive Cancer Center, Thomas Jefferson University Hospital, Philadelphia, Pennsylvania, USA; ^3^ Cancer Institute, Mass General Brigham Cancer Institute, Boston, Massachusetts, USA; ^4^ Department of Oncology, St Vincent’s University Hospital and Cancer Trials Ireland, Dublin, Ireland; ^5^ Department of Medical Oncology, West Virginia University, Morgantown, West Virginia, USA; ^6^ Department of Hematology/Oncology, Fox Chase Cancer Center, Philadelphia, Pennsylvania, USA; ^7^ Paediatric Oncology Unit, Fondazione IRCCS Istituto Nazionale dei Tumori, Milan, Italy; ^8^ Knight Cancer Institute, Oregon Health & Science University, Portland, Oregon, USA; ^9^ Samsung Medical Center, Sungkyunkwan University School of Medicine, Seoul, South Korea; ^10^ Department of Hematology, Oncology and Cancer Immunology, Charité – Universitätsmedizin Berlin, Berlin, Germany; ^11^ Seoul National University Hospital, Cancer Research Institute, Seoul National University College of Medicine, Integrated Major in Innovative Medical Science, Seoul National University Graduate School, Seoul, South Korea; ^12^ State Key Laboratory of Holistic Integrative Management of Gastrointestinal Cancers, Beijing Key Laboratory of Carcinogenesis and Translational Research, Department of Early Drug Development Centre, Peking University Cancer Hospital & Institute, Beijing, China; ^13^ Early Development Statistics Department, Evidenze Germany GmbH, Essen, Germany; ^14^ Clinical Development of Oncology, Bayer HealthCare Pharmaceuticals, Inc., Basel, Switzerland; ^15^ Clinical Development Pharmaceutical, Bayer S.p.A., Milan, Italy; ^16^ Thoracic Oncology Service, Memorial Sloan Kettering Cancer Center, New York, New York, USA; ^17^ Department of Medicine, Weill Cornell Medical College, New York, New York, USA; ^18^ Department of Investigational Cancer Therapeutics, The University of Texas MD Anderson Cancer Center, Houston, Texas, USA

**Keywords:** gene fusion, *NTRK1*, *NTRK3*, papillary thyroid carcinoma, thyroid cancer, TRK inhibitor

## Abstract

Larotrectinib is a first-in-class, highly selective, central nervous system-active tropomyosin receptor kinase (TRK) inhibitor approved for tumour-agnostic use in TRK fusion cancer. It has previously demonstrated rapid and durable disease control and favourable safety in patients with advanced TRK fusion thyroid carcinoma (TC). After an additional 4 years of follow-up with the inclusion of two additional patients, and utilising an independent review committee (IRC) to assess overall response rate (ORR), we report updated pooled analyses from three phase 1–2 larotrectinib clinical trials, focusing only on patients with TRK fusion differentiated TC (DTC). The primary endpoint was the IRC-determined ORR per RECIST v1.1. Duration of response (DoR), progression-free survival (PFS), overall survival (OS) and safety were also assessed. Twenty-four patients (papillary TC, *n =* 21; follicular TC, *n* = 2; poorly DTC, *n* = 1) were included (data cut-off: 20 July 2024). ORR was 79% (95% confidence interval (CI): 58–93); best responses were complete response in 3 (13%) patients, partial response in 16 (67%), stable disease in 3 (13%), progressive disease in 1 (4%) and not evaluable in 1 (4%). Median DoR and PFS were 35 (95% CI: 22–not estimable (NE)) and 44 (95% CI: 35–NE) months, respectively; 6-year OS rate was 71% (95% CI: 50–91). Six patients remained on treatment. Treatment-related adverse events (TRAEs) were mainly grade 1/2; no patients permanently discontinued treatment due to TRAEs. Larotrectinib continues to demonstrate durable disease control, extended survival and a favourable long-term safety profile in patients with advanced TRK fusion DTC requiring systemic therapy.

## Introduction

Tropomyosin receptor kinase (TRK) proteins are encoded by three distinct genes: *NTRK1*, *NTRK2* and *NTRK3*. This family of receptor proteins plays an important role in nervous system development and function (1, 2, 3). *NTRK* gene fusions are oncogenic drivers in various cancers, including follicular cell-derived thyroid carcinoma (3, 4). The overall prevalence of *NTRK* gene fusions in patients with thyroid carcinoma is estimated to be ∼2% (5). However, *NTRK* gene fusions are more common in papillary thyroid carcinoma (PTC), the predominant type of differentiated thyroid carcinoma (DTC), than in poorly differentiated thyroid carcinoma (PDTC) or anaplastic thyroid carcinoma (ATC) (6). *NTRK* gene fusions are also more commonly identified in tumours from paediatric patients compared with adults (16% vs 6%) (7). Thyroid carcinomas with TRK fusions most frequently involve *NTRK3* and *NTRK1*, with the *ETV6*::*NTRK3* fusion being the most prevalent (6, 7). While relatively low detection rates might discourage routine testing for *NTRK* gene fusions in thyroid carcinomas (7), the introduction of effective TRK-targeted therapies underscores its importance.

Larotrectinib is a first-in-class, highly selective, central nervous system (CNS)-active TRK inhibitor and is the first tumour-agnostic, molecularly targeted drug approved for solid tumours by the U.S. Food and Drug Administration (FDA) (8, 9, 10, 11). It is indicated for use in adult and paediatric patients with TRK fusion cancer and received full FDA approval in April 2025, after an initial accelerated approval was granted in November 2018 (8, 10, 12). The development programme for larotrectinib involved three clinical studies comprising patients of any age and any tumour type: a phase 1 study in adults (NCT02122913), a phase 1–2 study in children (SCOUT; NCT02637687) and a phase 2 ‘basket’ study in adolescents and adults (NAVIGATE; NCT02576431). Across these studies, larotrectinib demonstrated a rapid, robust and durable objective response in patients with various tumour types, leading to its approval (13, 14, 15, 16). As of July 2024, larotrectinib was associated with an independent review committee (IRC)-assessed overall response rate (ORR) of 65% across 304 patients with TRK fusion cancer (14).

In the subset of patients with TRK fusion thyroid carcinoma, larotrectinib has previously shown a rapid response, durable antitumour efficacy and a favourable safety profile in a small cohort, including both adult and paediatric patients with DTC and ATC (11). Integrated analysis of data from the subgroup of 21 patients with papillary or follicular DTC (including one case classified as PDTC) revealed an investigator-assessed objective response rate of 86% (95% confidence interval (CI): 64–97). In comparison, in ATC (including two cases of PDTC), single-agent larotrectinib resulted in an objective response rate of 29% (95% CI: 4–71). Treatment-related adverse events (TRAEs) were mostly grade 1 or 2.

Herein, we report updated integrated results in adult and paediatric patients with TRK fusion DTC treated with larotrectinib from three clinical trials. This analysis differs from the previously conducted study in that it includes two additional patients not included in the original analyses, incorporates IRC-assessed ORR and provides an additional 4 years of safety and efficacy data in this cohort of patients.

## Materials and methods

With a data cut-off date of 20 July 2024, this protocol-mandated analysis provides updated efficacy and safety data for larotrectinib in patients with TRK fusion DTC, compared with the previously published report (11), which had a data cut-off date of 20 July 2020. Full methods and eligibility criteria have been previously published (11, 16, 17, 18), so are described only briefly here.

### Study design and participants

This pooled analysis included data from patients with locally determined (i.e. per investigator) metastatic or locally advanced DTC harbouring an *NTRK* gene fusion. Patients were treated with larotrectinib in 1 of 3 clinical trials including adults (≥18 years old) with advanced solid tumours (NCT02122913), paediatric patients (<21 years old) with advanced solid or primary CNS tumours (SCOUT; NCT02637687) and adults and adolescents (≥12 years old) with advanced TRK fusion solid tumours (NAVIGATE; NCT02576431). *NTRK* gene fusions were detected by local molecular testing in Clinical Laboratory Improvement Amendments-certified or similarly accredited laboratories.

Adult patients received larotrectinib 100 mg twice daily, and paediatric patients received larotrectinib 100 mg/m^2^ twice daily (maximum 100 mg twice daily). Treatment continued until disease progression, withdrawal of the patient from the study or unacceptable toxicity. However, treatment could be continued beyond progression if the patient was judged by investigators to be clinically benefitting.

### Study endpoints

The primary endpoint was IRC-assessed ORR per Response Evaluation Criteria in Solid Tumors, version 1.1 (19). ORR was defined as the number of patients with complete response (CR) and partial response (PR) out of the total number of patients who received treatment. Secondary efficacy endpoints included duration of response (DoR), defined as the time from the start of the initial response (in patients with CR or PR) to the date of disease progression or death; progression-free survival (PFS), defined as the time from the first dose of larotrectinib treatment to the earliest date of documented disease progression or death; and overall survival (OS), defined as the time from the first dose of larotrectinib to death from any cause. Safety outcomes (i.e. treatment-emergent adverse events (TEAEs) and TRAEs) were also assessed.

### Study assessments

Tumours were assessed using a combination of computed tomography, magnetic resonance imaging and clinical measurement at baseline, every 8 weeks for 12 months and then every 12 weeks thereafter until disease progression. For paediatric patients in the SCOUT trial, tumour assessment was conducted every 6 months in the absence of disease progression after 2 years of treatment. All tumour responses were confirmed at least 4 weeks after the initial response. Adverse events (AEs) were graded per National Cancer Institute Common Terminology Criteria for Adverse Events, version 4.03 (20) and assessed from the date that informed consent was obtained until at least 28 days after the last dose of larotrectinib was administered.

### Statistical analysis

DoR, PFS and OS were estimated using Kaplan–Meier analysis, and 95% CIs were calculated using the Clopper–Pearson method. Median follow-up durations for DoR, PFS and OS were estimated using the reverse Kaplan–Meier method.

## Results

### Patients

A total of 24 patients with TRK fusion DTC were identified at the data cut-off of 20 July 2024, including 4 patients from the phase 1 study, 1 patient from SCOUT and 19 patients from NAVIGATE. Baseline demographics and clinical characteristics are shown in Table 1. The median age at study enrolment was 60 years (range: 6–80), 2 (8%) patients were children, and 17 (71%) were female. One patient (4%) with locally classified DTC had PDTC, and the remaining patients were classified as having PTC (*n* = 21 (88%)) or follicular thyroid carcinoma (*n* = 2 (8%)). Twelve patients (50%) had an Eastern Cooperative Oncology Group performance status of 0, 8 patients (33%) had a status of 1, 3 patients (13%) had a status of 2, and 1 patient (4%) had a status of 3 at baseline. Four (17%) patients had CNS metastases at baseline.

**Table 1:** Baseline patient demographics and clinical characteristics of 24 patients with TRK fusion DTC treated with larotrectinib.

Characteristic	All patients
	*n* = 24
Sex, *n* (%)	
Male	7 (29)
Female	17 (71)
Median age (range), years	60 (6–80)
Age group, *n* (%)	
<18 years	2 (8)
≥18 years	22 (92)
ECOG performance status, *n* (%)	
0	12 (50)
1	8 (33)
2	3 (13)
3	1 (4)
Tumour histology, *n* (%)^†^	
Papillary	21 (88)
Follicular	2 (8)
PDTC	1 (4)
*NTRK* gene fusion, *n* (%)	
*NTRK1*	11 (46)
*NTRK2*	0
*NTRK3*	13 (54)
*NTRK* fusion partner, *n* (%)	
*TPR::NTRK1*	5 (21)
*TPM3::NTRK1*	3 (13)
*DIAPH1::NTRK1*	1 (4)
*IRF2BP2::NTRK1*	1 (4)
*PPL::NTRK1*	1 (4)
*ETV6::NTRK3*	12 (50)
*EML4::NTRK3*	1 (4)
CNS metastases at baseline, *n* (%)	
Yes	4 (17)
No	20 (83)
Prior therapies, *n* (%)^‡^	
Surgery	24 (100)
Radioactive iodine	22 (92)
Radiotherapy	19 (79)
Systemic therapy^§^^,^^‖^	12 (50)
Number of prior lines of systemic therapy, median (range)^§^^,^^‖^	1 (0–5)
Number of prior lines of systemic therapy, *n* (%)^§^^,^^‖^	
Treatment naive^¶^	12 (50)
1	6 (25)
2	3 (13)
≥3	3 (13)
Best response to prior systemic therapy, *n* (%)^#^	
Stable disease	2 (8)
Progressive disease	3 (13)
Others^††^	10 (42)

^†^ Classified per the investigator.

^‡^ Patients may be counted in more than 1 row.

^§^ In the metastatic/unresectable setting.

^‖^ Prior systemic therapy comprised kinase inhibitors (lenvatinib (*n* = 7), sorafenib (*n* = 5), pazopanib and trametinib (each *n* = 2) and cabozantinib and sunitinib (each *n* = 1)) and immunotherapy (ipilimumab and pembrolizumab (each *n* = 1)).

^¶^ Patients were considered treatment naive if they had not received systemic therapy (excluding prior radioactive iodine) in the metastatic and/or unresectable settings.

^#^ Including responses to radioactive iodine and therapies in the non-metastatic setting.

^††^ Includes unknown, not evaluable and missing.

DTC, differentiated thyroid carcinoma; CNS, central nervous system; ECOG, Eastern Cooperative Oncology Group; PDTC, poorly differentiated thyroid carcinoma; TRK, tropomyosin receptor kinase.

There were seven unique gene fusions, with *NTRK1* and *NTRK3* gene fusions identified in 11 (46%) and 13 (54%) patients, respectively; there were no *NTRK2* gene fusions. The most common gene fusion was *ETV6*::*NTRK3* (*n* = 12 (50%)). *NTRK* gene fusions were identified by next-generation sequencing in all patients.

Prior treatments included radioactive iodine in 22 patients (92%), surgery in all patients and radiotherapy in 19 patients (79%). Twelve (50%) patients had received prior systemic therapy in the metastatic/unresectable setting, which could include kinase inhibitor therapy (cabozantinib, lenvatinib, pazopanib, sorafenib, sunitinib, trametinib) and immunotherapy (ipilimumab, pembrolizumab). Of these 12 patients, 6 (25%) received 1 prior therapy, 3 (13%) received 2 prior therapies, and 3 (13%) received ≥3 prior systemic therapies (Table 1). Three of the four patients with CNS metastases had received prior radiotherapy and/or surgery for their CNS metastases.

### Efficacy

**Figure 1:**
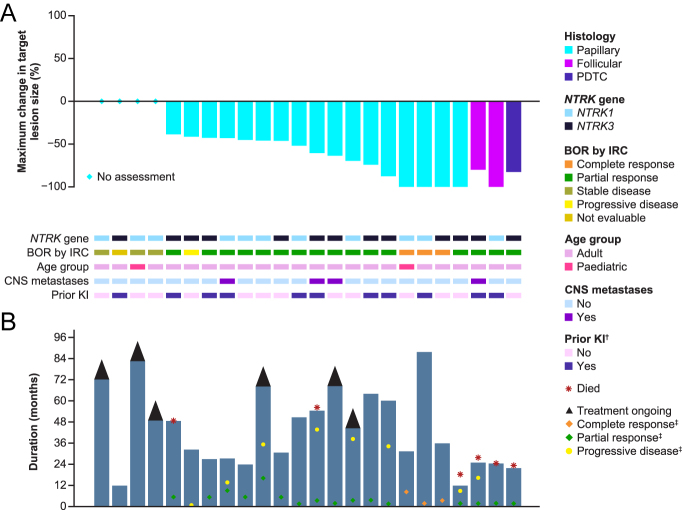
Tumour response in 24 patients with TRK fusion DTC. Combined waterfall and swimmer plots showing (A) maximum change in target lesion size following treatment with larotrectinib and (B) larotrectinib treatment duration. ^†^Comprised cabozantinib, lenvatinib, pazopanib, sorafenib, sunitinib, trametinib. ^‡^Indicates start of response, not BOR. BOR, best overall response; CNS, central nervous system; DTC, differentiated thyroid carcinoma; IRC, independent review committee; KI, kinase inhibitor; PDTC, poorly differentiated thyroid carcinoma; TRK, tropomyosin receptor kinase.

All patients with measurable disease at baseline as determined by IRC (*n* = 20, 83%) had tumour shrinkage. Of the four patients without tumour shrinkage, three had not received prior kinase inhibitor therapy (Fig. 1, panel A). The IRC-assessed ORR for all patients was 79% (95% CI: 58–93); 3 (13%) patients had a CR, 16 (67%) had a PR, 3 (13%) had stable disease (all ≥24 weeks of duration), 1 (4%) had progressive disease, and 1 (4%) was not evaluable due to non-measurable disease at baseline (Table 2). In the one patient with progressive disease, a brain lesion was noted in a follow-up scan; however, since there was no available baseline brain imaging, we were unable to confirm whether the presumed metastasis was new or pre-existing. All four patients with CNS metastases at baseline showed a best response of PR. Tumour responses were observed with larotrectinib administered in the first line as well as in later lines, with ORRs of 67% (95% CI: 35–90) in those with no prior systemic therapy (excluding radioactive iodine) in the metastatic setting, 83% (95% CI: 36–100) in those with 1 prior therapy and 100% (95% CI: 29–100) in those with 2 and ≥3 prior therapies (Table 2).

**Table 2:** Tumour response in 24 patients with TRK fusion DTC treated with larotrectinib, delineated by the number of prior systemic regimens.

	All patients (*n* = 24)	Number of prior systemic regimens^†^			
		Treatment naive^‡^ (*n* = 12)	1 (*n* = 6)	2 (*n* = 3)	≥3 (*n* = 3)
ORR, % (95% CI)	79 (58–93)	67 (35–90)	83 (36–100)	100 (29–100)	100 (29–100)
Best overall response, *n* (%)					
Complete response	3 (13)	2 (17)	1 (17)	0	0
Partial response	16 (67)^§^	6 (50)	4 (67)	3 (100)	3 (100)
Stable disease	3 (13)	3 (25)	0	0	0
≥24 weeks	3 (13)	3 (25)	0	0	0
Progressive disease	1 (4)	1 (8)	0	0	0
Not evaluable	1 (4)	0	1 (17)	0	0

^†^ Prior systemic therapy comprised kinase inhibitors (cabozantinib, lenvatinib, pazopanib, sorafenib, sunitinib, trametinib) and immunotherapy (ipilimumab, pembrolizumab).

^‡^ Patients were considered treatment naive if they had not received systemic therapy (excluding prior radioactive iodine) in the metastatic and/or unresectable settings.

^§^ Included 1 patient with PDTC (duration of response 22 months).

DTC, differentiated thyroid carcinoma; CI, confidence interval; ORR, overall response rate; PDTC, poorly differentiated thyroid carcinoma; TRK, tropomyosin receptor kinase.

In all patients, the median time to response was 1.9 months (range: 1.6–16.2) and the median duration of treatment was 40 months (range: 12–88). The time to best response ranged from 1.8 to 8.4 months in the three patients with a CR (aged 13, 65 and 74 years). At data cut-off, 6 patients (25%) remained on treatment, 4 of whom had ongoing clinical benefit (Fig. 1, panel B). Among patients who had discontinued treatment, 9 were alive without progression at the data cut-off (best responses were 3 CR, 5 PR and 1 not evaluable), 3 were alive with progressive disease and 6 had died (Fig. 1, panel B). Reasons for discontinuing treatment included physician decision (*n* = 4), patient disenrolment from the clinical trial (*n* = 3) and transition to commercially supplied larotrectinib (*n* = 2). All three patients with a CR had discontinued treatment and were alive without disease progression at their last evaluable tumour assessment.

No clear clinical risk factors for disease progression were identifiable, with no relationship discernible between duration of treatment and prior kinase inhibitor use or CNS metastases at baseline. Patients with follicular or poorly differentiated histologies had shorter duration of treatment than those with papillary histology; however, the small patient numbers limit any strong conclusions regarding histology and treatment duration (Fig. 1). Among 19 patients with available post-treatment circulating tumour DNA (ctDNA) samples, 6 patients had acquired resistance, 1 of whom had an acquired *GNAS* (p.R844H) pathogenic variant.

**Figure 2:**
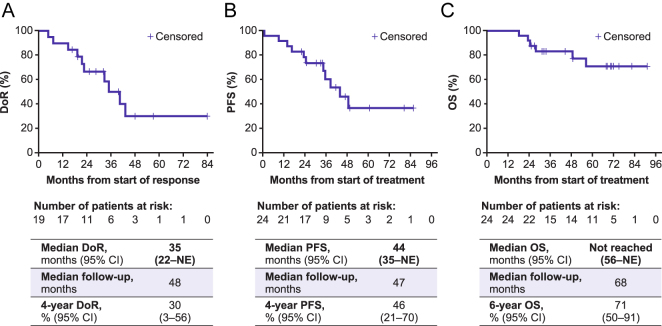
Kaplan–Meier plots of (A) IRC-assessed DoR, (B) IRC-assessed PFS and (C) OS in 24 patients with TRK fusion DTC treated with larotrectinib. CI, confidence interval; DoR, duration of response; DTC, differentiated thyroid carcinoma; IRC, independent review committee; NE, not estimable; OS, overall survival; PFS, progression-free survival; TRK, tropomyosin receptor kinase. A full colour version of this figure is available at https://doi.org/10.1530/ERC-25-0531.

After a median follow-up of 48 months, the Kaplan–Meier-estimated median DoR was 35 months (95% CI: 22–not estimable (NE)). The 4-year DoR rate was 30% (95% CI: 3–56; Fig. 2, panel A). Median DoR ranged from 29 to 84 months in the three patients with a CR. No evident association was observed between DoR and prior kinase inhibitor use, age, sex or *NTRK* gene fusion, but the sample size was insufficient to allow for definitive conclusions (Supplementary Fig. 1 (see section on Supplementary materials given at the end of the article)). Kaplan–Meier-estimated median PFS and OS were 44 months (95% CI: 35–NE) and not reached (95% CI: 56–NE) after median follow-up durations of 47 and 68 months, respectively (Fig. 2, panels A, C). The 4-year PFS rate was 46% (95% CI: 21–70) (Fig. 2, panel B), and the proportion of patients alive after 6 years was 71% (95% CI: 50–91) (Fig. 2, panel C).

### Safety

**Figure 3:**
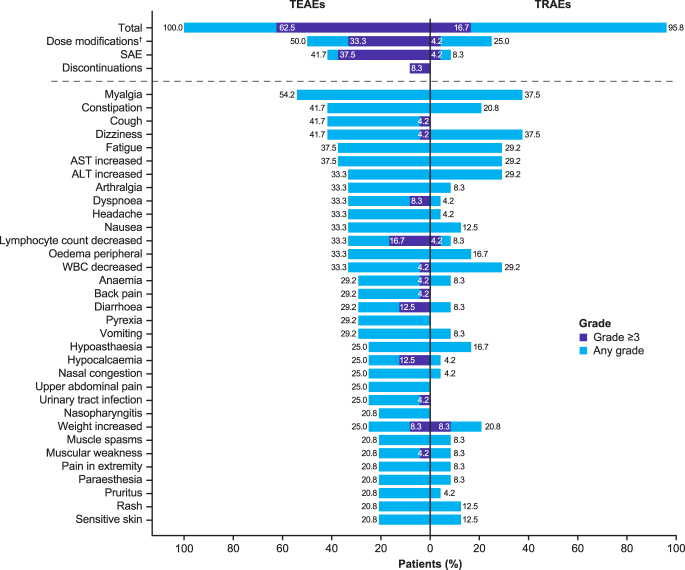
Safety profile of larotrectinib in 24 patients with TRK fusion DTC. Note: Only AEs reported in >20% of patients are shown. ^†^Includes dose hold/reductions and withdrawals. AE, adverse event; ALT, alanine aminotransferase; AST, aspartate aminotransferase; DTC, differentiated thyroid carcinoma; SAE, serious adverse event; TEAE, treatment-emergent adverse event; TRAE, treatment-related adverse event; TRK, tropomyosin receptor kinase; WBC, white blood cell.

TEAEs that occurred in >20% of patients are shown in Fig. 3. AEs were reported in all 24 (100.0%) patients, and TRAEs were reported in 23 patients (95.8%) (Fig. 3). Overall, TEAEs and TRAEs were grade 1/2 at worst in 9 (37.5%) and 19 (79.2%) patients, respectively. The worst severity of TEAEs and TRAEs was grade 3/4 in 12 (50.0%) and 4 (16.7%) patients, respectively.

Grade 3/4 TRAEs (all grade 3) were increased weight in two patients and decreased lymphocyte count and hepatic cytolysis in one patient each. In the patient with hepatic cytolysis, the grade 3 TRAE lasted for 16 days, prompting larotrectinib dose interruption. Larotrectinib was restarted at a lower dose when the event was grade 2, and the event eventually resolved.

Grade 5 TEAEs occurred in 3 (12.5%) patients, 2 of whom died of cancer progression and 1 from complications of infection. No deaths were attributed to larotrectinib treatment.

A total of 12 (50.0%) and 6 patients (25.0%) experienced a TEAE or TRAE leading to dose modification, respectively. TRAEs leading to dose modification included paraesthesia, increased aspartate aminotransferase and alanine transaminase levels, palmar-plantar erythrodysaesthesia syndrome, headache, fatigue, myalgia and hepatic cytolysis (each occurring in one patient). No patients permanently discontinued larotrectinib due to TRAEs.

## Discussion

First discovered in 1986 in colon carcinoma (21), *NTRK* gene fusions play an important role as oncogenic drivers in various malignancies in both children and adults (4). Although identified across various thyroid carcinoma histologies, *NTRK* gene fusions are most commonly associated with PTC, with an estimated prevalence of about 2% (5, 6). TRK fusion thyroid carcinoma is more common in younger vs older patients and is associated with invasive tumour characteristics (e.g. extrathyroidal extension, intravascular invasion and distant metastasis) (7, 22, 23). Single-centre studies have reported the prevalence of *NTRK* gene fusions in distantly metastatic PTC to be approximately 11% and 26% in adult and paediatric patients, respectively (24, 25), underscoring the importance of TRK fusions in the population of patients with advanced thyroid carcinoma who may need systemic therapy. The advent of therapies selectively targeting TRK has led to an evolutionary change in the management of TRK fusion cancers, including follicular cell-derived thyroid carcinomas.

In this current updated report on long-term outcomes in patients with TRK fusion DTC, larotrectinib treatment was associated with a high IRC-assessed response rate (ORR: 79% (95% CI: 58–93)), long durability (median DoR: 35 months (95% CI: 22–NE)), extended survival (median OS not reached (95% CI: 56–NE)) and a favourable safety profile without any new safety signals. The ORR in this study was consistent with the investigator-assessed objective response rate in the previous analysis of 28 evaluable patients with thyroid carcinoma (71% (95% CI: 51–87)), which included 7 patients locally characterised as having ATC (2 of whom had PDTC) (11). It was also similar to the objective response rate in the overall tumour-agnostic analysis (79% (95% CI: 72–85)) (17). With an additional 4 years of follow-up from the previous report and with the addition of an independent review of tumour response, the current analysis more fully characterises the durability of disease control with larotrectinib in this patient population. The extended median PFS/OS and the 6-year OS rate of 71% associated with larotrectinib are highly encouraging, further supporting the efficacy of larotrectinib in this patient population. In addition, the longer-term safety observed in the current analysis was in line with the previously reported safety profile of larotrectinib (11, 16, 17). TRAEs were mostly grade 1 or 2, and no patients permanently discontinued treatment due to a TRAE. While grade 3 treatment-related hepatic cytolysis was reported in one patient, the event was manageable with larotrectinib interruption and spontaneously resolved after resumption of larotrectinib at a reduced dose. This is in line with information given in the prescribing information, which acknowledges the potential for hepatotoxicity and provides guidance for its management, including withholding, reducing or permanently discontinuing larotrectinib dependent on event severity (8, 10).

The results also showed that larotrectinib was effective both as first-line therapy and following multiple lines of prior systemic treatment outside of radioactive iodine. This is important because patients with thyroid carcinoma often receive multiple therapies, and resistance to therapy is common (26, 27). Demonstrating larotrectinib efficacy after failure of other lines shows that it can be effectively used in heavily pre-treated patients for whom treatment options are lacking (26). The demonstration that larotrectinib is also efficacious as first-line therapy raises the possibility of using it upfront in TRK fusion thyroid carcinomas, potentially sparing patients the off-target toxicities of multikinase inhibitors (26, 27). An analysis of 101 treatment-naive patients with metastatic/unresectable TRK fusion cancer from the same three clinical trials also supported the efficacy and safety of first-line larotrectinib, with a slightly higher ORR (77% (95% CI: 68–85)) than seen in treatment-naive patients in the current analysis (67% (95% CI: 35–90)) (15). The seemingly lower response rate in the first line in the current study was possibly due to small sample sizes in some subgroups and requires further investigation.

Reported cases of acquired resistance to larotrectinib in patients with solid tumours have included both on-target mechanisms of resistance, involving secondary mutations in the TRK domain, and off-target mechanisms, involving alterations in bypass signalling pathways, for example *EGFR* amplification and *BRAF* alterations (3, 28, 29, 30). *NTRK* solvent-front mutations appear to be the main resistance mechanism occurring in patients with TRK fusion thyroid carcinoma (6). While the current analysis was not designed to evaluate resistance, we did detect an acquired *GNAS* (p.R844H) pathogenic variant in 1 of 6 patients with available post-treatment ctDNA samples and acquired resistance. Future studies with a focus on mechanisms of *a priori* and acquired resistance may help optimise treatment outcomes for patients treated with a selective TRK inhibitor. Another important future direction is the potential redifferentiation effect of larotrectinib (31, 32, 33), a concept currently being investigated in an ongoing clinical trial (NCT05783323).

An obvious limitation of the current analysis is the lack of direct active-comparator data; however, TRK fusion thyroid carcinoma is rare and effective targeted therapy options for advanced TRK fusion DTC are limited (34). In addition, the lack of central pathological review is a limitation of the study, with noted inaccuracies in local classifications of the included thyroid carcinomas (e.g. inclusion of PDTC in the PTC group and classification of some tumours as follicular thyroid carcinoma, which generally do not harbour TRK fusions) (5, 6). The strengths of this study include the long duration of follow-up and inclusion of both adult and paediatric patients.

In conclusion, with an additional 4 years of follow-up and an IRC-assessed ORR, treatment with larotrectinib for TRK fusion DTC in both first and subsequent lines of therapy results in durable disease control, extended survival and long-term tolerability without new safety signals, supporting the use of larotrectinib in patients with advanced TRK fusion DTC requiring systemic therapy.

## Supplementary materials



## Declaration of interest

SGW reports travel support and honoraria from Bayer. MSB reports research support from the University of Pennsylvania School of Medicine from Bayer, Loxo Oncology, Genentech, Eisai, Blueprint Medicines Corporation, Eli Lilly and Novartis; consultancy for Bayer, Loxo Oncology, Genentech, AstraZeneca and Eli Lilly; and honoraria from Clinical Care Options, Medscape, OncLive and PeerView. JJL reports compensated consultancy for Genentech, C4 Therapeutics, Blueprint Medicines Corporation, Nuvalent, Bayer, Elevation Oncology, Novartis, Mirati Therapeutics, AnHeart Therapeutics, Takeda, CLaiM Therapeutics, Ellipses, AstraZeneca, Bristol Myers Squibb, Daiichi Sankyo, Yuhan, Merus, Regeneron, Pfizer, Roche, Gilead, Janssen, Nuvation Bio, Eli Lilly, Gilead, Triana, Nuvectis and Turning Point Therapeutics; honorarium and travel support from Pfizer, Takeda, Merus and Bristol Myers Squibb; institutional research funds from Roche/Genentech, Hengrui Therapeutics, Turning Point Therapeutics, Neon Therapeutics, Relay Therapeutics, Bayer, Pfizer, Bristol Myers Squibb, Elevation Oncology, Linnaeus Therapeutics, Nuvalent, Eli Lilly, Novartis, Summit Therapeutics, Bicycle Therapeutics, Sutro and Triana. RM reports advisory board participation with Amgen, Bayer, Bristol Myers Squibb, Clovis, Janssen and Pfizer; invited speaker activity for Astellas, Ipsen and MSD; local PI roles for Astellas, Bayer, Bristol Myers Squibb, Clovis and Regeneron Pharmaceuticals, Inc.; and a coordinating PI role for MSD. MA has nothing to disclose. JB reports advisory board participation with Pfizer, Bayer, Kura, Janssen, Blueprint Medicines Corporation, Merck, BeiGene, Turning Point Therapeutics, Merus and Fortvita and consultancy for Pfizer, AstraZeneca and Eli Lilly. MC reports advisory roles for AstraZeneca/Alexion, Bayer, Pfizer, Servier, Merck, Norgine and Roche. SK reports consultant/advisory board participation for Springworks Therapeutics, SeaGen, Bayer, Genome & Company, HarbourBioMed, BPGbio Therapeutics, Oxford Biotherapeutics, Mundibiopharma, BPGbio, Inc., Gilead, Mirati, GI Innovation Inc, XYone Therapeutics, Genome Insight, Aadi Biosciences, MOMA Therapeutics, Daiichi Sankyo, Parabilis, Boehringer Ingelheim Pharmaceuticals, PATHOMIQ (co-founder), Fortress Biotech, Inc (equity) and Sift Biosciences (equity). Their spouse is a scientific advisor for Cadila Pharmaceuticals, Ltd., and founder of Arxeon, Inc. S-HL reports serving a consulting or advisory role for Abion, AstraZeneca, BeiGene, Bristol Myers Squibb, Daiichi Sankyo, Eli Lilly, IMBdx, ImmuneOncia, Janssen, Merck, MSD, Novartis, Pfizer, Roche and Takeda; receiving honoraria from Amgen, AstraZeneca/MedImmune, Bristol Myers Squibb, MSD, Roche and Yuhan; and receiving research funding from AstraZeneca, Lunit and MSD, outside of the current work. DTR reports advisory roles for Bayer and BeiGene; travel support from Bayer and Johnson & Johnson; honoraria from Eli Lilly, Roche and Bristol Myers Squibb; and research support from Seagen. D-YO reports advisory/consultancy for AstraZeneca, Novartis, Genentech/Roche, Merck Serono, Bayer, Taiho, ASLAN, Halozyme, Zymeworks, Celgene, BeiGene, Basilea, Turning Point Therapeutics and Yuhan and research grant/funding (personal) from AstraZeneca, Novartis, Array, Eli Lilly, Servier, BeiGene, MSD and Handok. CQ has nothing to disclose. NN is an external employee of Bayer. D-IB is an employee of Bayer. CEM is an employee of Bayer. AD reports honoraria from 14ner/Elevation Oncology, Amgen, AbbVie, AnHeart Therapeutics, ArcherDX, AstraZeneca, BeiGene, BerGenBio, Blueprint Medicines, Bristol Myers Squibb, Boehringer Ingelheim, Chugai Pharmaceutical, EcoR1, EMD Serono, Entos, Exelixis, Helsinn, Hengrui Therapeutics, Ignyta/Genentech/Roche, Janssen, Loxo/Bayer/Eli Lilly, Merus, Monopteros, MonteRosa, Novartis, Nuvalent, Pfizer, Prelude, Regeneron Pharmaceuticals, Inc., Repare RX, Springer Healthcare, Takeda/Ariad/Millenium, Treeline Biosciences, Turning Point Therapeutics, Tyra Biosciences, Verastem and Zymeworks; advisory board participation with Bayer, MonteRosa, AbbVie, EcoR1 Capital, LLC, Amgen, Helsinn, Novartis, Loxo/Eli Lilly, AnHeart Therapeutics, Bristol Myers Squibb and Nuvalent; consulting with MonteRosa, Innocare, Boundless Bio, Treeline Biosciences, Nuvalent, 14ner/Elevation Oncology, Entos, Prelude, Bayer, Applied Pharmaceutical Science, Bristol Myers Squibb, Enlaza, Pfizer, Roche/Genetech, Novartis, Two River and Eli Lilly/Loxo; associated research paid to institution from Foundation Medicine, GlaxoSmithKlein, Teva, Taiho and PharmaMar; equity with mBrace and Treeline Biosciences; copyright with selpercatinib–osimertinib (filed/pending); royalties from Wolters Kluwer and UpToDate; other funding (food/beverage expenses) from Merck, Puma and Boehringer Ingelheim; and CME honoraria with Answers in CME, AXIS, Clinical Care Options, Doc Congress, EPG Health, Harborside Nexus, I3 Health, Imedex, Liberum, Medendi, Medscape, Med Learning, MedTalks, MJH Life Sciences, MORE Health, Ology, OncLive, Paradigm, PeerView Institute, PeerVoice, Physicians Education, Projects in Knowledge, Resources, Remedica Ltd, Research to Practice, RV More, Targeted Oncology, TouchIME and WebMD. DSH reports research/grant funding from AbbVie, Adaptimmune, Amgen, AstraZeneca, Bayer, Bristol Myers Squibb, Daiichi Sankyo, Eisai, Fate Therapeutics, Genentech, Genmab, Ignyta, Infinity, Kite, Kyowa, Eli Lilly, Loxo Oncology, Merck, MedImmune, Mirati, MiRNA, Molecular Templates, Mologen, NCI-CTEP, Novartis, Pfizer, Seattle Genetics and Takeda; travel/accommodation/expenses from Loxo Oncology, MiRNA, ASCO, AACR, SITC and Genmab; consulting/advisory roles for Alpha Insights, Axiom, Adaptimmune, Baxter, Bayer, Genentech, GLG, Group H, Guidepoint Global, Infinity, Janssen, Merrimack, Medscape, Numab, Pfizer, Seattle Genetics, Takeda and Trieza Therapeutics; and ownership interests in Molecular Match (advisor), OncoResponse (founder) and Presagia Inc (advisor). MEC reports research support from Bayer, Exelixis, Ignyta, Loxo Oncology, Blueprint Medicines Corporation, Eisai, Merck, Novartis and Genentech.

## Funding

These studies were funded by Bayer Healthcare and Loxo Oncology, Inc., a wholly owned subsidiary of Eli Lilly and Company. Dr Drilon was supported by the National Cancer Institute/National Institutes of Health grants P30CA008748 and 1R01CA226864-01A1 and Nonna’s Garden.

## Author contribution statement

SGW, MSB, JJL, RM, MA, JB, MC, SK, S-HL, DTR, D-YO, CQ, AD, DSH and MEC conceived the study; performed investigation; designed the methodology; and wrote, reviewed and edited the manuscript. D-IB and CEM conceived the study; designed the methodology; supervised the study; administered the project; and wrote, reviewed and edited the manuscript. NN conceived the study; designed the methodology; curated the data; performed formal analysis, investigation and validation; and wrote, reviewed and edited the manuscript.

## Data sharing statement

Availability of the data underlying this publication will be determined according to Bayer’s commitment to the EFPIA/PhRMA ‘Principles for responsible clinical trial data sharing’. This pertains to scope, time point and process of data access. As such, Bayer commits to sharing, upon request from qualified scientific and medical researchers, patient-level clinical trial data, study-level clinical trial data and protocols from clinical trials in patients for medicines and indications approved in the United States (US) and European Union (EU) as necessary for conducting legitimate research. This applies to data on new medicines and indications that have been approved by the EU and US regulatory agencies on or after 1 January 2014. Interested researchers can use www.vivli.org to request access to anonymised patient-level data and supporting documents from clinical studies to conduct further research that can help advance medical science or improve patient care. Information on the Bayer criteria for listing studies and other relevant information is provided in the member section of the portal. Data access will be granted to anonymised patient-level data, protocols and clinical study reports after approval by an independent scientific review panel. Bayer is not involved in the decisions made by the independent review panel. Bayer will take all necessary measures to ensure that patient privacy is safeguarded.

## Ethics statement

Study protocols were approved by the institutional review board or independent ethics committee at each site. The study was conducted according to International Ethical Guidelines for Biomedical Research Involving Human Subjects, Good Clinical Practice guidelines, the Declaration of Helsinki and all relevant local laws. All patients were required to provide written informed consent prior to study entry.
