# Supplementary material for: Larotrectinib in TRK fusion differentiated thyroid carcinoma: updated trial data
Source: Endocr Relat Cancer. 2026 Aug 5;33(8):e250531. doi: 10.1530/ERC-25-0531 (PMC13452024; doi:10.1530/ERC-25-0531)
Supplement: Supplementary file 1 [file ERC-25-0531_supplementary_figure_1.pdf]

Supplementary Materials

Supplementary Figure 1 Kaplan–Meier plots of effect of A) prior kinase therapy, B) age, C) sex and D) *NTRK* gene fusion on IRC-assessed DoR in 24 patients with TRK fusion differentiated thyroid carcinoma treated with larotrectinib.

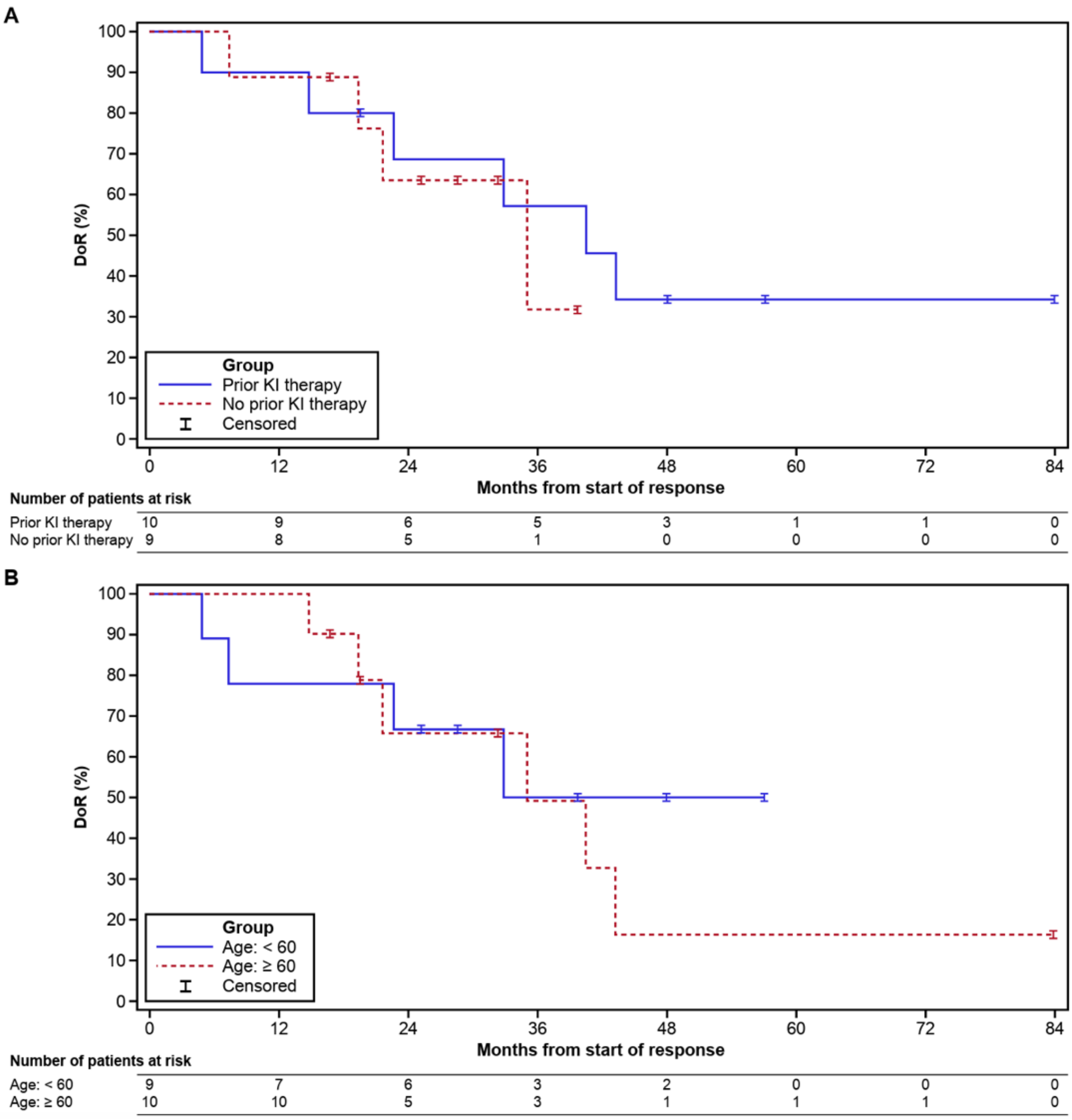

C

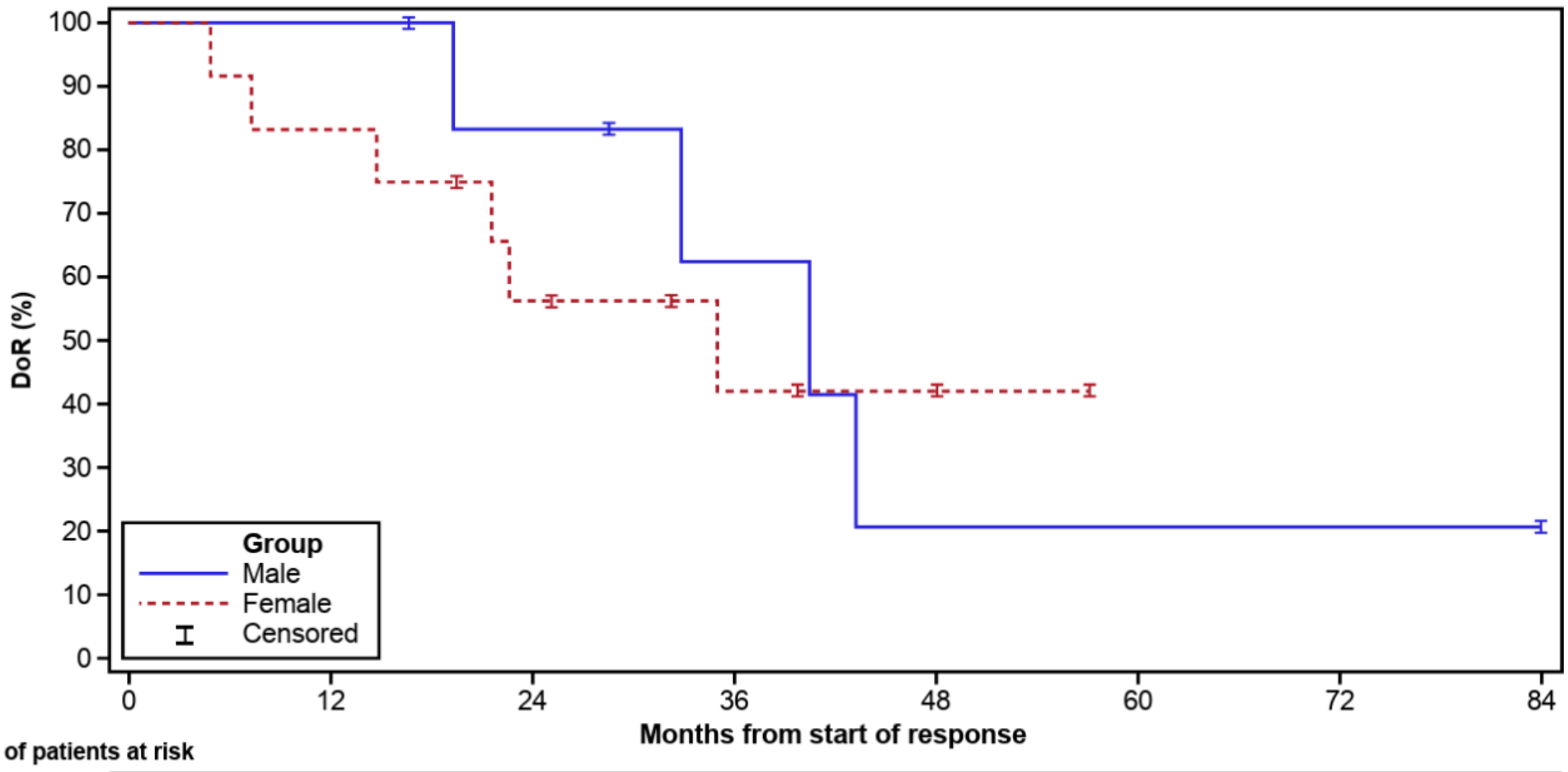

Number of patients at risk

Male	7	7	5	3	1	1	1	0
Female	12	10	6	3	2	0	0	0

D

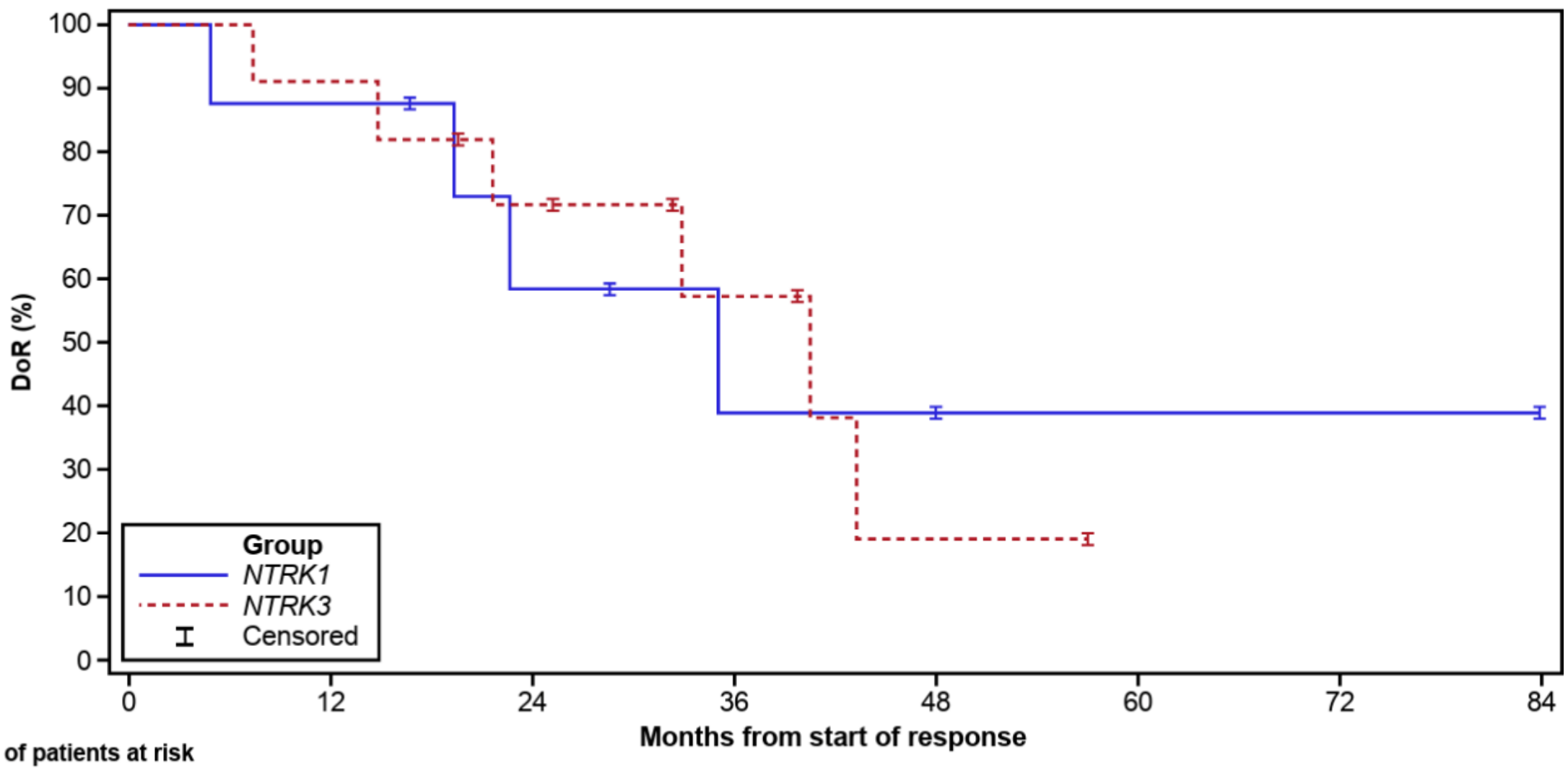

Number of patients at risk

NTRK1	8	7	4	2	2	1	1	0
NTRK3	11	10	7	4	1	0	0	0

DoR, duration of response; IRC, independent review committee; KI, kinase inhibitor.
